# An adaptation model for trabecular bone at different mechanical levels

**DOI:** 10.1186/1475-925X-9-32

**Published:** 2010-07-02

**Authors:** He Gong, Dong Zhu, Jiazi Gao, Linwei Lv, Xizheng Zhang

**Affiliations:** 1 Department of Engineering Mechanics, Jilin University, Changchun, 130025, China; 2 Department of Orthopedic Surgery, No. 1 Hospital of Jilin University, Changchun, 130021, China; 3 Institute of Medical Equipment, Academy of Military Medical Sciences, Tianjin, 300161, China

## Abstract

**Background:**

Bone has the ability to adapt to mechanical usage or other biophysical stimuli in terms of its mass and architecture, indicating that a certain mechanism exists for monitoring mechanical usage and controlling the bone's adaptation behaviors. There are four zones describing different bone adaptation behaviors: the disuse, adaptation, overload, and pathologic overload zones. In different zones, the changes of bone mass, as calculated by the difference between the amount of bone formed and what is resorbed, should be different.

**Methods:**

An adaptation model for the trabecular bone at different mechanical levels was presented in this study based on a number of experimental observations and numerical algorithms in the literature. In the proposed model, the amount of bone formation and the probability of bone remodeling activation were proposed in accordance with the mechanical levels. Seven numerical simulation cases under different mechanical conditions were analyzed as examples by incorporating the adaptation model presented in this paper with the finite element method.

**Results:**

The proposed bone adaptation model describes the well-known bone adaptation behaviors in different zones. The bone mass and architecture of the bone tissue within the adaptation zone almost remained unchanged. Although the probability of osteoclastic activation is enhanced in the overload zone, the potential of osteoblasts to form bones compensate for the osteoclastic resorption, eventually strengthening the bones. In the disuse zone, the disuse-mode remodeling removes bone tissue in disuse zone.

**Conclusions:**

The study seeks to provide better understanding of the relationships between bone morphology and the mechanical, as well as biological environments. Furthermore, this paper provides a computational model and methodology for the numerical simulation of changes of bone structural morphology that are caused by changes of mechanical and biological environments.

## Background

Bone is a living organ; it has the ability to adapt to mechanical usage or other biophysical stimuli in terms of its mass and architecture. This attribute is known as functional adaptation [1,2]. Modeling by drifts and remodeling by groups of osteoclasts and osteoblasts that are organized into basic multicellular units (BMUs) determine both the bone mass and architecture, with the exception of longitudinal bone growth. Bone modeling works best during the growing years and works poorly on adult cortical bone. However, it works satisfactorily within trabeculae throughout life [3]. Bone remodeling consists of biologically coupled BMU activation, bone resorption by osteoclasts, and bone formation by osteoblasts. It occurs in all in vivo bone tissues and is an important way to renew bone.

The observed adaptations of bone mass and architecture to the mechanical usage show that a certain mechanism exists for monitoring the mechanical usage and controlling bone adaptation behaviors. A conceptual "mechanostat" theory developed in a previous study indicates that the mechanically generated bone strain signals have important roles in governing bone adaptation behaviors [4,5]. Threshold ranges of such signals appear to reside in certain skeletal cells as genetically determined internal standards. Minimum effective strain (MES) has been used to describe the above internal standards (or "thresholds") [4]. Three thresholds, i.e., remodeling, modeling and microdamage thresholds (MESr, MESm and MESp, respectively) serve as boundaries of four zones describing different bone adaptation behaviors: the disuse, adaptive, overload, and pathologic overload zones.

Bone adaptation process is controlled by mechanical usage and biological factors, which have coupled contributions [6]. Aging, menopause, drug treatments, and so on, are known as biological factors. Researchers found that biological factors regulate the rate of bone turnover; however, the overall balance between bone formation and resorption is influenced by prevailing levels of mechanical usage [7].

Nowadays, heavy computing power has been mastered by human beings, making the quantitative simulation of bone adaptation process possible; this is driven by the goals of further predicting and explaining the bone adaptation behaviors, as well as the formation and maintenance of bone architecture under different mechanical and estrogen levels. In doing so, the method of conducting actual experiments has limited efficacy since certain difficulties existed in some experimental investigations. Simulation saves not only time, but also expenditures spent on research and development, it is also regarded as 'the third way of science' [8]. Many attempts have been made to gain quantitative insight into the bone modeling and remodeling processes:

Researchers begin with theoretical analyses before conducting numerical simulation on bone structures in cooperation with the finite element method. One of the fundamental theories ("theory of adaptive elasticity") for cortical bone was based on general continuum mechanical principles [9]. Based on this theory, a finite element model has been developed [10]. A different approach to predict bone adaptation behavior postulated that bone is a self-optimizing material able to adapt its orientation and density in response to its stress/strain state [11]. In another remodeling algorithm, the idea of a lazy or homeostatic zone has been included [12]. The essential idea is that bone mass increases above a certain level of strain or strain energy density; in addition excessive bone remodeling can be observed below a certain threshold. In between the two levels, the bone structure is maintained, representing the case when bone is under physiological loading conditions encountered in normal activities [13]. The idea of homeostatic zone is similar with the adaptation zone proposed by Frost [4,5]. There are also a number of alternative remodeling algorithms that have been proposed and used in previous study [14-16]. All trabecular models simulated the outcome of coordinated osteoclastic and osteoblastic activities as either a net increase or decrease in density. A model has been developed that included separate descriptions of osteoclastic resorption and osteoblastic formation, enabling simulation of trabecular bone growth, adaptation and maintenance [17,18]. It was assumed that osteocytes can transfer an osteoblast recruitment stimulus to the surface and then enhance bone formation; in addition, osteoclasts could resorb bone that is in disuse state or has been damaged in a spatially random manner [17]. In addition, using six bone resorption models, Tanck et al. investigated how using alternative strain-based local stimuli for osteoclasts in bone resorption would affect remodeling and adaptation of trabecular architecture [19]. Vahdati and Rouhi added the cellular accommodation effect into the model and took into consideration both microdamage and disuse on activation of resorption [20]. Nowadays, increased computational resources have made it possible for large-scale bone-remodeling simulations to have an element size that is as small as about 50 με. This demonstrates that bone remodeling at the tissue level can create a highly complex and optimized trabecular structure in terms of bone density and orientation [21-23].

In the current quantitative bone adaptation models, the amount of osteoblastic bone formation is proportional to the difference between the mechanical stimulus and a certain threshold (setpoint). According to Frost's mechanostat theory, the changes of bone mass in different zones, as calculated by the difference between the amount of bone formation and that of bone resorption, should be different, resulting in different bone adaptation behaviors. This means that the coupling relationship between bone formation and resorption may be different. In this paper, an adaptation model for trabecular bone at different mechanical levels is proposed based on a number of experimental observations and numerical algorithms in the literature, in which the amount of bone formation and the activation of bone adaptation behaviors have been proposed in accordance with the mechanical levels. It is convenient to determine which zone a bone surface lies in as well as calculate the changes of its mass. Hence, more insights about the relationships between bone morphology and the mechanical/biological environments may be gained.

## Methods

This section presents an adaptation model for trabecular bone at different mechanical levels. Seven numerical simulation cases under different mechanical conditions are then analyzed as examples by incorporating our adaptation model with the finite element method on a simplified two-dimensional finite element model of a trabecular bone.

### Effects of strain energy density (SED) on osteoblastic formation and the probability of osteoclastic activation

Osteocytes act as sensors of mechanoreceptors and regulators of bone mass by mediating the actor cells (osteoblasts and osteoclasts) [24,25]. In mathematical models governing the bone adaptation process, the strain energy density has been suggested as the mechanical signal that the osteocytes appraise [12]. The total mechanical stimulus at location *x *on the trabecular surface (*P*(*x*,*t*)) is the contribution of all the osteocytes made in the forms of SED, relative to their distance from *x *[26]:(1)

where *μ*_*i *_is the mechanosensitivity of the osteocyte *i*, *R_i_(t) *is the SED of the osteocyte *i*, and *f*_*i*_(*x*) is the spatial influence function, which describes the influence of osteocyte *i *on the osteoblasts and osteoclasts at location *x *[15]:(2)

where *d*_*i *_(*x*) is the distance between osteocyte *i *and location *x*. The parameter *D *represents the distance from an osteocyte, at which location the effect has been reduced to 0.36788.

There are about 1600 osteocytes per square millimeter. An osteocyte is located in the center of each bone-filled element [27]. The influence parameter *D *was equal to 100 *μm *[15].

For the osteoclastic activation, experimental and numerical studies show that apart from disuse-activated resorption, microdamage-stimulated resorption also correlates with the SED value in bone tissue, i.e., accumulated osteocyte signal [20,28].

Based on the aforementioned investigations, several SED thresholds were used to represent different mechanical levels for osteoblast formation, as well as the probability of osteoclast activation at the tissue level. Then, the adaptation model for the trabecular bone at different mechanical levels was presented, resulting in different adaptation behaviors at the apparent level as described by Frost's mechanostat theory [4,5]. The symbols and expressions of the SED thresholds are listed in Table 1.

**Table 1:** Symbols and expressions of the SED mechanical thresholds, and the values in our numerical simulations

Symbol	Expression	Value in numerical simulation (MPa)
*K*_*RD*_	Threshold for resorption drift of bone modeling	0.0357
*K*_*OB*_	Osteoblastic formation threshold	0.0714
*K*_*AD*1_	Lower threshold for the adaptive zone	0.0918
*K*_*AD*2_	Upper threshold for the adaptive zone, and also the threshold for formation drift of bone modeling	0.1122

### Osteoclast activation and bone resorption

The amount of bone resorbed by osteoclasts at a trabecular surface patch is denoted as *r*_*oc*_, where the affected surface patches is determined by chance (Huiskes et al. [17]). These authors introduced a probability of osteoclast activation per surface site at any time, which is assumed to be regulated either by the presence of microcracks or by disuse. Vahdati and Rouhi [20] proposed that the probability of microdamage-stimulated resorption is a quadratic function of the accumulated osteocyte signal, and that below a critical load value, microdamage would not initiate remodeling, thus include a region similar to the lazy zone. Based on the aforementioned investigations, the probabilities of osteoclast activation per surface site at different mechanical levels, i.e., in different mechanical zones are described below.

(1) In the disuse zone, the probability of osteoclast activation was regulated by disuse. The response of the probability of the osteoclast activation to the osteocyte signals is set to be sigmoidal, similar to the responses found in pharmacological applications [29].

(2) Under physiological loading conditions encountered in normal activities, the probability of osteoclast activation is equal for all surface sites. This assumption is based on the observations that remodeling continuously renews the skeleton daily, and that the dynamic forces of daily living produces microcracks. The distribution of microcracks has been found to be spatially random [17,30].

(3) When overloaded, the probability of osteoclast activation is assumed to be regulated by microdamage as the first phase of remodeling to repair damaged regions. Thus, the quadratic function of the accumulated osteocyte signal, as suggested by Vahdati and Rouhi [20], is used to describe the probability of osteoclast activation in this zone.

Thus the resorption probability (*p*(*x*,*t*)) as a function of stimulus value in the form of SED (*P*(*x*,*t*)) can be expressed as:(3)

where *p*_max_, *a*, *b*, *c*, *d*, and *g *are constants. In the numerical examples presented in this paper, the values of these constants are *p*_max _= 0.2, *a*= 90.0, *b*= 0.0714, *c*= 0.0275, *d*= 176.0, and *g *= 19.506.

**Figure 1:**
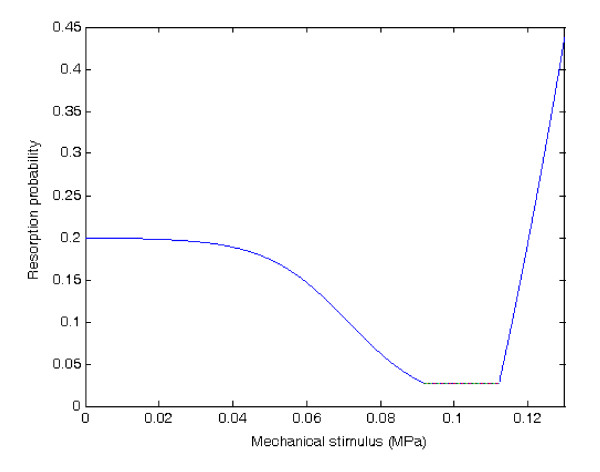
**Relationship between resorption probability and mechanical stimulus in the numerical simulation**.

These parameter values have been chosen as follows: since *c *represents the resorption probability in the adaptive zone, it has been given a small value of 0.0275. For the resorption probability in the disuse zone, the form is sigmoidal and the maximum value is *p*_max_; it has been set to 0.2 [20]. Parameters *a *and *b *are coefficients that define the slope and inflection point of the curve, respectively. The inflection point has been chosen as *K*_*OB*_, and *a *is determined from the condition of continuity at *P*(*x*,*t*) = *K*_*AD*1_. The resorption probability is a quadratic function when overloaded. By assuming the resorption probability of 0.25 when *P*(*x*,*t*) = 0.122436 MPa and that the condition of continuity is at *P*(*x*,*t*) = *K*_*AD*2_, the values of *d *and *g *can thus be obtained. As a result, the resorption probability is 1 when *P*(*x*,*t*) = 0.149 MPa. For a large range of mechanical stimulus from 0 to 0.149 MPa, the average resorption probability was 0.2148, which is of the order of trabecular bones that are regenerated per year [17]. The plot of resorption probability as a function of stimulus value is shown in Figure 1.

When the mechanical stimulus remains lower than *K*_*RD*_, i.e., the threshold for resorption drift of bone modeling, the bone surface is subject to resorption by osteoclasts alone, which is the case for bone resorption drift in the bone modeling process.

### Osteoblast recruitment and bone formation

Osteoblast recruitment at a surface site is determined by the formation stimulus in the form of SED. The proposed amount of bone formation per surface site (*r*_*ob*_) at different mechanical levels is expressed below:

(1) If the total stimulus is less than the osteoblast formation threshold, bone formation will not occur.(4-1)

(2) If the stimulus is higher than the osteoblast formation threshold (*K*_*OB*_), but is lower than the lower threshold for the adaptive zone (*K*_*AD1*_), bone formation will occur at the surface site. This is proportional to:(4-2)

where *τ*_1 _is constant.

(3) When the stimulus is between the two thresholds for the adaptive zone, the amount of bone formed per surface site (*r*_*ob*_) will be equal to the amount of bone resorbed (*r*_*oc*_):(4-3)

resulting in no net change of bone mass in the adaptive zone.

For Equations (4-2) and (4-3), under the condition of continuity, *r*_*ob *_= *r*_*oc *_when *P*(*x,t*) = *K*_*AD*1_, thus .

(4) If the stimulus is higher than the higher threshold for adaptive zone (*K*_*AD*2_), bone formation at the surface site will be proportional to:(4-4)

where *τ*_2 _is a proportionality constant that had been chosen as *τ*_2 _= 1 MPa^-1 ^in the numerical simulation presented in this paper [31].

**Figure 2:**
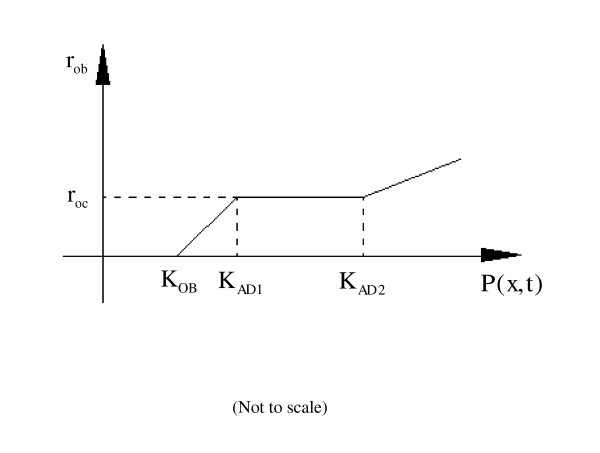
**Schematic diagram of the relationship between the amount of bone formation (*r*_*ob*_) and the mechanical stimulus *P*(*x,t*)**.

Figure 2 shows the schematic diagram of the relationship between the amount of bone formation (*r*_*ob*_) and the mechanical stimulus *P*(*x,t*).

The local change of the relative bone density *m *(1.0 for fully mineralized tissue) in location *x *can be expressed as:(5)

The Young's modulus *E*(*x,t*) at each location is density-dependent:(6)

where *E*_max _was the maximum tissue level Young's modulus: *E*_max _= 500 *MPa *[32], and *r *= 3 [33].

### Numerical approach - a simplified two-dimensional finite element model of a trabecular bone

**Figure 3:**
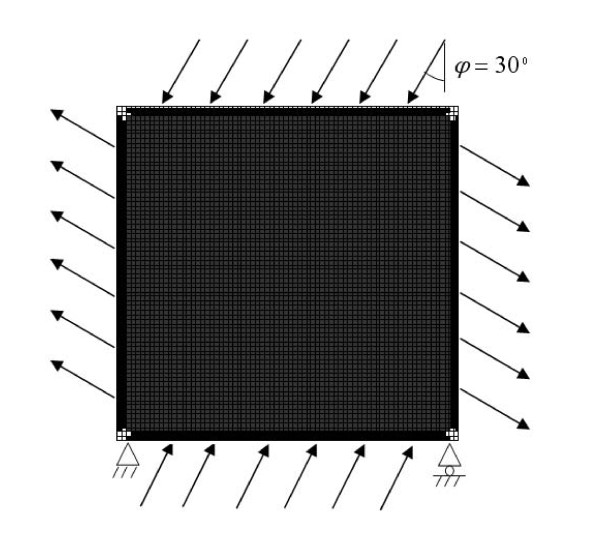
**The initial finite element model**. The 2 mm × 2 mm portion of bone volume was meshed with 80 × 80 four-node bilinear elements, representing the bone adaptation area; side plates with 2-element thicknesses were added to the bone volume to create even deflection at the edges, which did not remodel.

The bone adaptation simulation was performed on a simplified two-dimensional finite element model of a 2 *mm *× 2 *mm *portion of bone volume with a thickness of 0.02 *mm*; it was loaded at the edges by a tensile and compressive principal stress with a uniform density distribution of *m*(*x,t*_0_) = 0.7, in which *t*_0 _represented the beginning of the simulation (Figure 3). The orientation of the principal stress was φ = 30°. The bone plate model was meshed with 80 × 80 four-node bilinear elements. Boundary conditions were considered important since only a small volume of bone was to be analyzed. Side plates with 2-element thicknesses were added to the bone volume to create even deflection at the edges. The side plates were of constant maximal density and did not remodel. As suggested in [31], surfaces close to the edge of the mesh, particularly surface corners, have a reduced osteocytic environment to receive stimuli compared with the elements located in the centre of the mesh. A correction of stimulus was made by a factor in the regions close to the edge of the mesh, as shown in [31]. The structure was loaded by a sinusoidal stress cycling between 0 and 2 MPa at 1 Hz; it can be calculated using the static finite element analysis [31]. The static finite element analysis combined with the bone remodeling equations proposed by Zhu et al. [16] was performed using the ANSYS software to obtain the homeostatic architecture of the bone model. The volume fraction of this homeostatic architecture was 0.613, similar to that obtained in [31]. Mechanical strain in the bone tissue was about 1041 με, which was within the adaptive zone defined by the mechanostat theory of Frost [3,4]. Hence, the reference value for mechanical stimulus in this simulation was chosen as the average of the two SED thresholds defining the adaptive zone, i.e., *K*_*AD*1 _and *K*_*AD*2_, in the numerical simulations that follow. The values for the SED thresholds in Table 1 are chosen so that the numerical results in this paper may agree with the experimental observations.

The homeostatic architecture served as the starting point for the following simulation cases. The computer simulation was conducted as an iterative process in computer time, in which the relative density *m *per element was regulated between 0.01 and 1.0 (Eq. (5)). The bone adaptation scheme for the 2 D plate model was performed using the ANSYS Parametric Design Language (APDL). Each surface site was given a stochastic number by random function in the APDL for every iterative time step. If the stochastic number was smaller than the resorption probability *p*(*x,t*) in Eq. (3) for the same surface site, this site will be subjected to osteoclastic resorption. Once the osteoclasts were activated in a surface site, they resorbed a fixed amount of bone tissue with a relative density of 0.38, which was derived from the following aspects. The area within an osteonal cement line was 2.84 × 10^-2 ^*mm*^2 ^[34]. However, in the trabecular bone, BMUs resorbed and refilled trenches rather than tunnels; hence, the bone remodeling area was assumed to be 1.42 × 10^-2 ^*mm*^2^. The resorption period was about 60 days [35]. The area resorbed by osteoclasts was 2.367 × 10^-4 ^*mm*^2^/day. Each finite element area in the model was 6.25 × 10^-4 ^*mm*^2^, indicating that a relative density of 0.38 was resorbed by osteoclasts.

The trabecular architecture in the following cases was simulated by incorporating the proposed bone adaptation model with the finite element method. This was done to verify whether the proposed bone adaptation simulation method is capable of simulating the adaptation behavior of the trabecular bone at different mechanical levels.

### Simulation Cases

#### Simulation 1: Disuse

**Figure 4:**
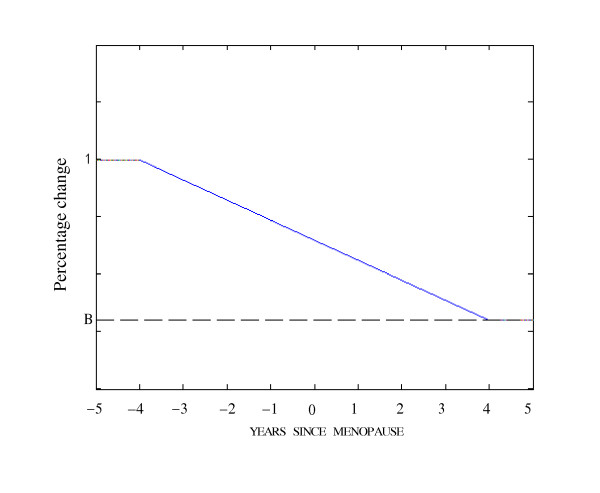
**Pattern of disuse**. The mechanical loadings decreased according to the curve to simulate mechanical disuse. The values on the curve show the percentages in the mechanical loadings to their magnitudes for the homeostatic structure. *B *describes the percentage of mechanical loadings in the end of menopause.

The mechanical loading condition of disuse was applied to the homeostatic architecture within a period of eight years (four years before menopause and four years after menopause). The mechanical loading on the homeostatic model decreased according to the curve in Figure 4 to simulate mechanical disuse [2]. The percentage change in mechanical loading was chosen so that the predicted change in bone mineral density (BMD) will correspond with the clinical data that measures the lumbar spine BMD every six months throughout the menopausal transition by Recker et al. [36].

**Figure 5:**
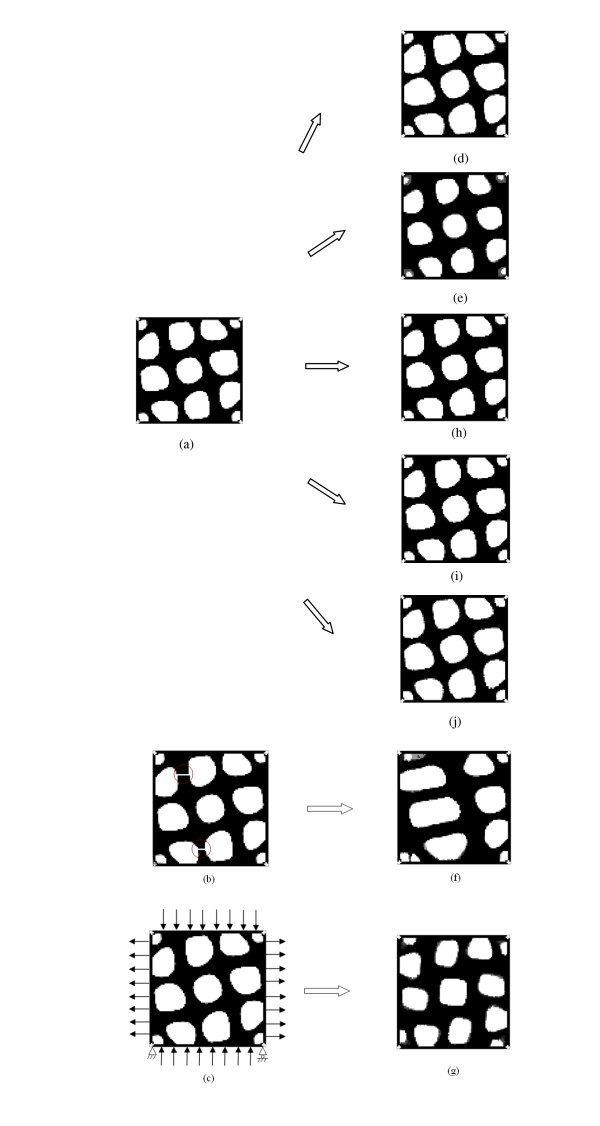
**The homeostatic architecture of the bone model, the initial architectures and loading conditions of the seven simulation cases, and their simulation results**. **a **The homeostatic structure of the bone model, which is the initial architecture of simulations 1, 2, 4, 5, 6 and 7. **b **The initial architecture of simulation 3: two struts were artificially disconnected (inside the two circles), while the external stress was maintained. **c **The initial architecture and loading condition of simulation 4. The initial structure was the homeostatic structure. The orientation of the applied stress was changed from 30° to 0°. **d **Result of simulation 1. **e **Result of simulation 2. **f **Result of simulation 3. **g **Result of simulation 4. **h **Result of simulation 5. **i **Result of simulation 6. **j **Result of simulation 7.

Figure 5(a) shows the homeostatic structure of the bone model, which is the initial architecture of simulations 1, 2, 4, 5, 6 and 7.

#### Simulation 2: Overloading

A 20% increase in the magnitude of the mechanical loading was imposed on the homeostatic structure to investigate the effect of overloading.

#### Simulation 3: Artificially disconnected trabeculae

Two struts in the homeostatic architecture were artificially disconnected, while the same externally applied loads were maintained (Figure 5(b)), as proposed by Vahdati and Rouhi [20].

#### Simulation 4: Rotation of the external load

The orientation of the applied stress on the homeostatic architecture was changed from 30° to 0° (Figure 5(c)).

#### Simulation 5: Some increase in the external load within physiological loading condition

In this simulation, an 8% increase in the magnitude of the mechanical loading was imposed on the homeostatic structure.

#### Simulation 6: Some decrease in the external load within physiological loading condition

Here, an 8% decrease in the magnitude of the mechanical loading was imposed on the homeostatic structure.

#### Simulation 7: Effect of menopause

In this simulation, the mechanical thresholds in Table 1 were arbitrarily increased by 20%, given that the objective of this simulation case was to gain qualitative insight of the effect of menopause. This would result in the rightward movement of the resorption probability curve in Figure 1, which qualitatively means that the resorption probabilities were minutely increased for the mechanical stimuli originally in the adaptive zone, and even more increased for those originally in disuse condition. The mechanical loading condition was the same as that used in simulation 6.

There are numerous physiological bases for this simulation case. It is well known that bone loss at menopause is associated with estrogen deficiency. Researchers found that estrogen directly acts on osteoclasts and regulates the lifetime of osteoclasts [37]. Bone resorption is regulated by both the number of osteoclasts and the activity of each osteoclast. In another bone-remodeling simulation scheme, the bone loss patterns associated with menopause, similar to the clinical observations, have been obtained by increasing the birthrate of new BMUs over the perimenopausal period [38,39]. Moreover, Nyman et al. increased the mechanostat set point over 3 years to simulate the effect of estrogen withdrawl that produces bone loss observed during menopause [40]. Based on the above investigations, the number of osteoclasts appears to be a more dominant parameter regulating bone resorption, compared with the activity of each osteoblast at menopause. In this study, by increasing the mechanical thresholds in the proposed adaptation model, the increase of resorption probability at menopause can be simulated.

## Results

**Figure 6:**
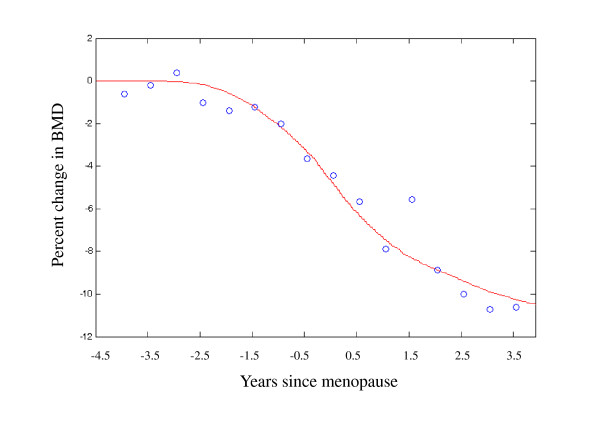
**Percent changes in bone mass from 4 years before, to 4 years after menopause**. The data points show the clinical data measured by Recker et al. [36]. The curves representing percent changes in bone mass are the simulated outcomes. A 20% end reduction in the external loading (*B *= 0.8 in Figure 4) leads to the lowest sum of the squared error (SSE) from the clinical data.

Figures 5(d) to Figure 5(j) show the predicted trabecular architectures for simulations 1 to 7. Figure 5(d) is the numerical result for simulation 1, i.e., disuse mechanical loading condition. Figure 6 gives the percent changes in bone mass from 4 years before, to 4 years after menopause. A 20% end reduction in the external loading (*B *= 0.8 in Figure 4 is shown to reduce trabecular thickness to a loss of bone mass by 10.48%. The clinical and simulated data are highly correlated (*R*^2 ^= 0.95). The sum of the squared error (SSE) is 11.68. Hence, our simulation results are consistent with the bone loss patterns clinically observed at menopause [36].

In simulation 2, increasing the load amplitude by 20% gradually thickened the trabeculae (Figure 5(e)). As a result, the bone mass increased by 16.45%. Accordance is observed with several experiments showing that high-impact gymnastics or stronger muscles increases bone mass [41,42].

In the case of artificially disconnected trabeculae (simulation 3), two struts in the homeostatic architecture were artificially disconnected (Figure 5(b)). As a result of modeling and remodeling, the unloaded trabeculae disappeared and the adjacent ones thickened with little change in bone mass (Figure 5(f)).

The direction of loads applied to the homeostatic architecture changed in simulation 4 (Figure 5(c)). After rotating the external loads from 30° to a perpendicular orientation, the trabeculae gradually realigned to adapt to the new loading directions (Figure 5(g)).

In the next two simulations, we investigated the bone adaptation behaviors under physiological loading conditions. Visibly, an 8% increase in the external loads only caused 3.12% increase in bone mass with little change in trabecular architecture (Figure 5(h)). Similarly, an 8% decrease in the external loads only resulted in 1.33% decrease in bone mass. The trabecular architecture changed insignificantly (Figure 5(i)).

**Figure 7:**
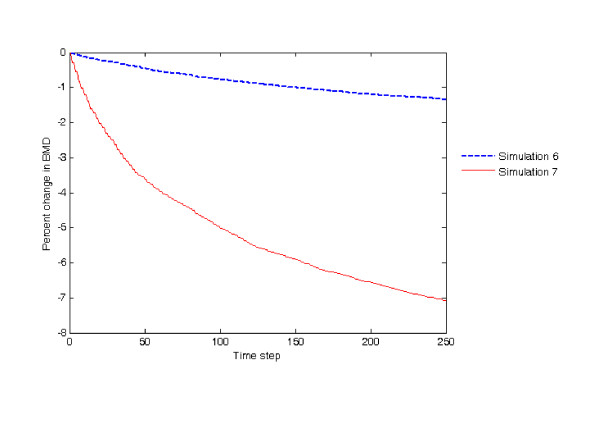
**Percent changes in bone mass for simulations 6 and 7, respectively**.

In the final simulation, the effect of menopause on the mechanical thresholds was investigated. Given that most aging adults lose momentary muscle strength and the intensity of their physical exercise becomes weaker [43], the external loads on the homeostatic architecture were the same with those in simulation 6, i.e., the external loads fell toward the lower end of physiological loading condition. For the mechanical thresholds, a 20% increase resulted in a 7.08% decrease in bone mass. Figure 7 shows the percent changes in bone mass for simulations 6 and 7, from which the effect of mechanical thresholds on bone mass may be clearly seen. The numerical outcome of simulation 7 is shown in Figure 5(j).

## Discussion and Conclusion

The objective of this paper is to understand the contributions of mechanical loading and menopause on the adaptation behaviors of trabecular bone. Here, we developed a numerical bone adaptation model based on bone physiology, in which the amount of bone formation and bone remodeling activation were in accordance with the mechanical levels.

We investigated our bone adaptation algorithm using seven simulation cases, and confirmed that the algorithm is able to predict the adaptation behaviors of trabecular bone under different mechanical conditions. In this study, the formulations on the amount of bone formation and bone remodeling activation probability were proposed based on a number of experimental and numerical investigations. For bone remodeling activation probability, Vahdati and Rouhi [20] suggested that bone remodeling is governed by SED when loading was below a certain threshold value, and by damage when loading exceeded the threshold. In the bone metabolic model of Huiskes et al. [17], the probability of osteoclast activation is considered to be regulated, either by the presence of microcracks within the bone matrix caused by the dynamic forces of daily life, or by disuse since osteoclast activation is enhanced by lack of loading. Tanck et al. [19] investigated the effects of osteoclastic resorption characteristics on trabecular bone remodeling. We proposed the characteristics of bone remodeling activation at different mechanical levels: in disuse zone, the probability of osteoclast activation was controlled by disuse; under physiological loading conditions, it was equal for all surface sites; and in overload zone, it was assumed to be regulated by microdamage. For bone formation, there was more bone formation in highly strained areas, which was similar to the previous numerical algorithms, but the relationship between the amount of bone formation and the mechanical stimulus was not taken as a unique linear form, but a sectioned linear form. The phenomenon that there was no change of bone mass under physiological loading conditions in normal activities was also taken into account. This model could be extended to simulate age-related bone loss. The rate of age-related bone loss is generally between 0.3% and 1.1% per year [44]. In our bone adaptation model, the amount of formed bone can be modeled as a little bit less than the amount of bone resorbed by osteoclasts, resulting in the so-called formation deficit.

In our numerical implementations, the computer simulation was conducted as an iterative process in computer time, in which the relative density per element was regulated between 0.01 and 0.1 (Eq. (5)). The number of iterations was not the actual time (e.g., in the unit of days), but the relationship between the number of iterations and the actual time could be estimated from the beginning and end equilibrium bone masses (see Figure 6 for example) [16]. All simulation results are consistent with the clinical observations and experimental investigations. Hence, by incorporating the proposed bone adaptation model with finite element analysis, trabecular bone adaptations can be simulated at different mechanical levels.

**Figure 8:**
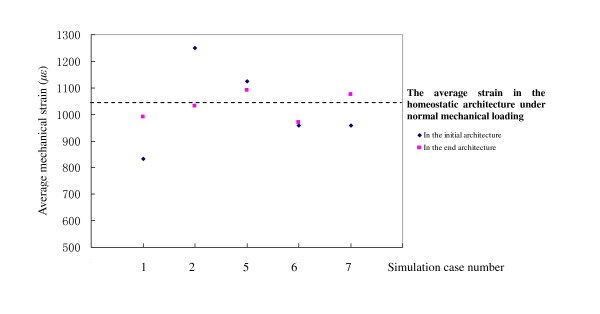
**The average mechanical strains of bone elements in the initial and end trabecular architectures for simulations 1, 2, 5, 6, and 7 under their mechanical loading conditions, respectively**.

The simulated bone adaptation mechanism is also in accordance with the mechanostat theory of Frost, who proposed that the mechanostat is a biological mechanism that fits skeletal mass and architecture to the needs of normal physical activities of an individual. The mechanical loading made the system correct errors between the mass and its loading. The behaviour of the mechanism is similar to a home thermostat, which would turn "on" in response to error and turn "off" in its absence, hence, the name "mechanostat" [4,5,45]. In the present numerical study, the initial trabecular architecture for simulations 1, 2, 5, 6, and 7 was the homeostatic architecture (Figure 5(a)), which was not suitable to the mechanical and/or biological conditions in those simulation cases. This is why the bone adaptation processes have been initiated. Figure 8 shows the average mechanical strains of bone elements in the initial and end trabecular architectures for simulations 1, 2, 5, 6, and 7 under their mechanical loading conditions, respectively. The average mechanical strain in the homeostatic architecture (Figure 5(a)) under its normal mechanical loading was 1041*με*. When the load decreased by 20% (simulation 1), the average strain in this architecture decreased to 833 *με*, which was far from that in the homeostatic environment, so the bone adaptation algorithm removed a few bone material, until the average strain rose to 990 *με*, i.e., the average strain in the new architecture (Figure 5(d)) went back to the adaptive zone. When the external load increased by 20% (simulation 2), the average strain in the homeostatic architecture increased to 1250 *με*; thus, the bone adaptation algorithm continued to add bone material, until the average strain fell to 1031*με *to make the new architecture (Figure 5(e)) return to the adaptive zone. Due to the existence of the so-called lazy zone, i.e., a physiological range in which alteration of strain will produce no significant adaptation response, an 8% increase or decrease of external load in simulations 5 and 6, respectively, caused little change of architecture (Figures 5(h) and 5(i)) and strain (Figure 8). Significant attention should thus be given to the comparison between simulations 6 and 7. The external loads in these simulations were the same, but the mechanical thresholds were increased due to menopause in simulation 7. As a result, the average strain in the end configuration (Figure 5(j)) increased by 10.72%, in comparison with simulation 6 (Figure 5(i)).

A number of limitations associated with this study may have contributed to our simulations. In the proposed adaptation model, the adaptation behaviors in the pathological overload zone were not included, because in this case, bone tissue showed nonlinear mechanical properties until fracture, which requires further study using nonlinear and fracture mechanics. Another limitation was that the adaptation processes were simulated on a 2 D model, therefore, estimations of adaptation for 3 D samples of bone cannot be obtained. Despite of the limitations mentioned above, the adaptation model for trabecular bone at different mechanical levels proposed in this paper has a number of potential applications. The model may help us gain a better understanding of the relationships between bone morphology and the mechanical, as well as biological environments. Recently, state-of-the-art computational techniques in both hardware and software have been utilized to handle millions of finite elements in the PC base (even hundreds of millions in the supercomputer base) [21-23]. Hence, a further prospect of the bone adaptation model proposed in this paper is to perform a detailed 3 D bone-remodeling simulation. By incorporation with finite element method, the adaptation of bone structure to the mechanical and biological stimuli, e.g., vertebral body, proximal femur, and tibia, which are trabeculae rich skeletal sites that are most at risk of bone loss in elderly population, can be simulated.

Furthermore, this paper provides a methodology for the numerical simulation of changes of bone structural morphology caused by changes of mechanical environment under different circumstances such as orthopaedic surgery, bone internal and external fixations, artificial joints or other implants, as well as changes of biological environment caused by aging, menopause, or pharmacological therapies for osteoporosis. Usually, under such circumstances, bone morphology will be changed to adapt to the new mechanical and biological environments. Nevertheless, approximately more than half a year is required to observe such changes in clinics. Evaluations may then be made as to whether these changes are beneficial for the bone in the long run. To simulate the long-term bone morphological changes by incorporating bone adaptation model with finite element analysis is efficient, thereby decreasing the research period for the related problems and save a large amount of experimental expenditures. Given that the changes of mechanical and/or biological environments will induce some bone tissue to switch to other zones with different adaptation behaviors, our bone adaptation model would be fundamental to such applications.

## Competing interests

The authors declare that they have no competing interests.

## Authors' contributions

HG carried out the study design, the numerical simulations, and drafted the manuscript. DZ helped to perform the study design and draft the manuscript. JG participated in the numerical simulations. LL participated in the study design and manuscript draft. XZ coordinated the study, participated in the study design and manuscript draft. All authors read and approved the final manuscript.
